# Clinic of Jefferson Medical College

**Published:** 1844-11-16

**Authors:** 


### CLINICAL LECTURES AND REPORTS.

### PHILADELPHIA HOSPITAL.

#### CLINIC OF JEFFERSON MEDICAL COLLEGE.

*November* 9, 1844.

The first lectures of this clinic were delivered to a larger concourse of students than were ever seen within the walls of the extensive amphitheatre. Twenty-five omnibuses conveyed them from the city to the Hospital. Not fewer than five hundred were present; and as a clinic was held at the University of Pennsylvania, and the lectures of the Professors there commenced at 12 o'clock on the same morning, it is manifest, that the number of students in the city must be larger, perhaps, than at any former period of the history of medical instruction in Philadelphia.

At ten o'clock, Professor Pancoast commenced his lectures on Clinical Surgery, and operated before the class for False anchylosisof the lower jaw—Amputation of the great toe—Excision of the posterior part of the os calcis for caries—and opening a deep-seated abscess in the perineum;—accompanying his operations by appropiate remarks.

For the following full report of Professor Dunglison's lecture we are indebted to Mr. White, of Georgia, one of the students of the College, who favoured us frequently with reports of the same clinic last year.

#### CLINIC OF PROFESSOR DUNGLISON.

Professor Dunglison commenced this, the first, lecture of the course, by remarking, that the subject he had to teach in this place was clinical medicine—in lectures and demonstrations—not necessarily delivered, as the term clinical would signify, at the bedside, for it would be impracticable with so large an attendance of students,—but by presenting to the class in the amphitheatre such cases as were possessed of the greatest interest. He stated, that he would also be able, during the winter, to exhibit numerous pathological specimens, illustrative of the cases brought before them.

Clinical instruction he regarded as all important. It was, at present, attracting a larger share of attention in the medical world than at any previous period; and every endeavour was made by the schools of Philadelphia to render it as effective as possible.

Before entering on the subject proper of the lecture, Professor D. considered it well to make a few statements in regard to the existing arrangements for clinical instruction at the Hospital. He remarked, that for many years the lectures were confined entirely to professors of the University of Pennsylvania; but that soon after his appointment as one of the attending physicians, the Board of Guardians, desirous of increasing the scheme of clinical instruction, requested of the Medical Board of the Hospital to report to them whether this were practicable. The Board recommended that two days in the week should be set apart for this purpose;—one for the medical officers of the institution connected with the University of Pennsylvania; and the other for those connected with the Jefferson Medical College. The recommendation of the Medical Board was adopted, and the wards were divided between the officers attached to the two medical institutions. In this manner two schools were provided for; but a third—the Pennsylvania Medical College—was not represented by any medical officers. The students of this College, Professor Dunglison stated, were entitled to the same privileges as those attached to the others; and, for one, he would gladly welcome them there, should they feel disposed to attend his clinic.

This Hospital the Professor considers, in point of size, number of patients, and advantageous arrangements, to be second to none in America, and to few in Europe.

Having concluded his preliminary observations, Professor Dunglison proceeded to make some general remarks on the different modes of investigating disease; or, in other words, of forming a correct diagnosis.

He considers it important, in every case, to pay particular attention to the vital and physical manifestations, or (as some writers on semeiology express it) to symptoms and signs; by the former meaning those phenomena dependant on functional aberration; by the latter, those physical alterations of texture that are appreciable by the senses—of hearing and of touch more especially. Properly, every *symptom* is a *sign* of disease; yet there is convenience in the division into vital phenomena and physical signs. Exclusive attention to either of these is to be sedulously avoided. It is only by a careful association of the vital with the physical manifestations, that clear and correct ideas of the diseased condition can be obtained. The disposition to direct the attention exclusively to one class of phenomena has existed in every age of medicine, and nothing is more to be deprecated.

Pathological anatomy, by confirming or disproving our diagnosis, affords much aid in discovering the causes and symptoms of many affections, but it would be injudicious to confine the attention solely to it. In the same way, although the use of percussion affords invaluable assistance, its results are often, when taken alone, equivocal. To each and every morbid manifestation, the attention of the observer should be directed. But although it is judicious thus to combine and compare the evidences afforded by these various means of forming a judgment, as to the character of the affection in any case that presents itself, it is not always necessary; for it sometimes happens, that the vital manifestations are alone sufficient to discover clearly the nature of the malady.

In illustration of this point, the lecturer introduced a patient, with tumefaction of the wrist, resulting from sub-acute rheumatism. This, he observed, is at once recognized as a case of œdema or local dropsy (in contradistinction to anarsarca or general dropsy, and like all œdematous accumulations is dependant on a loss of balance, between the exhalant and absorbent vessels of the part. In the normal relation of these vessels, the absorbents take up what the exhalants deposit, and there is no acccumulation of fluid in the cellular membrane; but if the relation be destroyed, a collection of the secretion results, giving rise to the tumefied appearanc of the part, as exhibited in this case. The vital phenomena of dropsy are here evident, and are sufficient to establish the nature of the affection.

This case also serves to illustrate certain physical phenomena indicated by the sense of touch, which are easily comprehended. Thus, by pressing on the swelling, as the lecturer did with his finger, and then removing the finger, a depression is left. This results from the fluid contained in the portion of the cellular member under the finger being forced into the neighbouring cells—if they may be so termed. In a short time, the depression or pit disappears, in consequence of the reflux of the fluid to its former position.

The *vital manifestations* in disease are referable to several sources. In every case of diseased action when affecting the whole system, morbid functional phenomena, dependent upon disturbed circulation, innervation or secretion, are observable. When either of these great functions is disordered, the others are apt to be more or less affected. In almost every case, the function of secretion is disturbed, and it is with the view of detecting the extent of this disturbance, that the physician inspects the tongue. This is done, not to notice merely the changes that have taken place in the mucous membrane of the tongue, for they would afford unimportant information, but to judge from the appearance of its surface as to the degree in which the secretory system is implicated. When the secretions, with which its surface is covered, are greatly altered in character, the inference is, that a corresponding disturbance exists in the secreting organs in general. But from this indication alone, no just ideas as to the nature of the alteration can generally be deduced.

Again, the circulation is so constantly disordered in disease, that the pulse is almost always examined. What idea do we form from this examination? The pulsations of the radial artery are not felt with the view to determine merely the state of that vessel; but to learn the greater or less action of the central organ of the circulation. By it, the force, frequency, rhythm, &c., of the heart's contractions are determined. If it be frequent and forcible, excitement is present in the system; and, moreover, we are able to judge, as to the amount of blood circulating in the vessels, the degree of arterial tension, &c. The artery at the wrist is selected for observation, mainly on account of its convenient position. But—the professor remarked—the pulse alone is not to be implicitly relied on as an index of disease. He believed it to be, when taken singly—in the language of Celsus—*res fallacissima.* Other circumstances are to be considered along with it, and then much light may accrue from its careful examination. In proof of the equivocal results, from the consideration of the pulse alone, the lecturer related an instructive case—that of a patient who, in consequence of an amputation, had secondary hemorrhage which well nigh drained his vessels. By judicious measures the hemorrhage was arrested, and re-action in a short time fairly established. At this moment, a medical colleague of the surgeon in attendance—an old practitioner—entered the room, and feeling the patient's pulse—without any knowledge of what had happened—exclaimed:—"why does not my colleague bleed this person?" Here was an instance in which the pulse of reaction, after almost fatal hemorrhage, so much resembled, on cursory examination, that of inflammatory excitement, that a respectable physician, ignorant of the case, deemed it proper to bleed. In such cases as these,—the Professor took occasion to state,—a full dose of opium allays the erethism of the nervous system induced by excessive loss of blood, and restores peace and harmony where, before, all was confusion and turmoil. Such cases show the impropriety of deciding from an examination—and especially from one that is imperfect—of any single phenomenon; for although by careful attention, the pulse of inflammatory excitement, and that depending on erethism of the nervous system produced by copious bleeding, occurring spontaneously or by art, may be discriminated, yet it may lead astray the young practitioner. In every case, therefore, it is indispensable to the formation of correct pathological deductions to combine an investigation of all the vital manifestations.

The *physical signs*, as before stated, mean those changes which are appreciable by the senses, of hearing and palpation more especially. This mode of investigating disease is of comparatively recent date. The principles of percussion were first applied by Auenbrugger in the last century. Those of auscultation were introduced in the present century by Laënnec. Although the principle of both auscultation and percussion is extremely simple, some have denied that any information can be derived from them; yet the slightest knowledge of Acoustics is sufficient for their comprehension, and for the establishment of their utility.

[Here the Professor, for the better elucidation of the subject, sketched a diagram on the blackboard, to exhibit the chest and a portion of its contents.]

The thorax, formed of more or less elastic parietes, contains two organs of respiration—the lungs, into the composition of which so little solid matter enters, that, for all practical purposes, the thoracic cavity may be considered, except where the heart is situate, as occupied solely by air. Such being the normal condition of the parts, it can readily be conceived that percussion on its parietes would elicit a clear sound—and this is found to be the fact. The sound is clear or tympanitic—not tympanitic, however, in the ordinary auscultatory acceptation of the term—which is generally used to signify an unusual drum-like clearness on percussion, such as occurs in emphysema of the lungs. This being the effect of percussion, in the natural state of the chest and of its contents—the alteration, in the sound rendered on percussion, that would be produced by the introduction of solid matter, to occupy a space before almost solely filled by air, can easily be imagined. If percussion be made over a portion of lung consolidated by the deposition of tubercular matter in phthisis, or or of fibrin in pneumonia, the sound is flat—as would be anticipated. In applying, however, this principle, great caution is requisite to guard against error.

The sound is not the same over every part of the thorax, but it ought to be so, or nearly so, over corresponding portions of the opposite sides. In percussing the chest, therefore, the sounds should be compared on the same parts of the two sides, and if these vary materially, a deviation from the natural state may be suspected.

Again, when the ear is applied over the chest, in the healthy state of its contents, the sound of the air passing into the chest is distinctly audible. This mode of examination is called Auscultation. It also is employing, in conjunction with other senses, that of hearing in the study of disease.

This sound or murmur of respiration varies in different parts of the chest; being alike, or nearly so, however, in corresponding parts, so that as a matter of course, any marked difference in these situations would lead to the suspicion of disease. During health, the intensity is of a certain degree in various parts of the lung, but this may be materially modified by disease. Thus, in cases of consolidation of the tissue of the lung, in certain portions, by tubercular or other deposits, it is reasonable to infer, that there may be a diminution or total absence of the murmur of respiration; and this is found in practice to be actually the case.

Again, the voice in the natural condition of the parts passes downwards through the bronchial tubes, and resounds with a certain intensity throughout the chest. This resonance is propagated to the parietes by means of the pulmonary tissue, so as to be distinctly audible when the ear is applied over the chest. When, however, portions of the lung take on diseased action, this sound is modified. Thus, if there be solidification of its tissue, the conducting power thereof is increased in proportion to its amount—as solid bodies convey sound better than gaseous; and an increase in the intensity of the vocal resonance thus becomes an index of pulmonary consolidation.

By the association of these various modes of exploring disease by physical manifestations, great certainty can often be attained in the diagnosis of thoracic affections. Thus, take the example already noticed of consolidation of the lung, and we find that its existence is conclusively proved, when there are present the three physical phenomena just mentioned—dulness on percussion, diminution or extinction of the respiratory murmur, and increase in the vocal resonance. How beautifully do these harmonize? With what degree of reasonableness can any one deny the aids to diagnosis thus obtained? Yet there are still those who condemn and reject both percussion and auscultation as useless and even ridiculous innovations.

The lecturer next referred to the value of an attention to morbid anatomy, to which also, he remarked, too exclusive attention had been paid even of late years. He regarded it as a most valuable aid to diagnosis. The physical signs indicate to us the morbid anatomy of the organs whilst the patient is alive: dissection, of course, reveals only the lesions that are obtainable from the dead. To this important aid to diagnosis, and in a lesser degree to therapeutics, he proposed to direct the attention of the clinical class on every available opportunity.

Besides the modes already alluded to, the lecturer proceeded to mention others; some of which were derived from an examination of the secretions; others from the effects of remedies. Dr. Marshall Hall had pointed out and urged that the effect of certain remedies might be employed as a means of diagnosis in doubtful cases. His remarks principally apply to the effect of blood-letting in inflammatory and other diseases,—by noticing the degree of tolerance of the system to the abstraction of blood, this tolerance being great in cases of high inflammatory action, and generally bearing some proportion to it; while, on the other hand, but little tolerance denotes that the excitement is to a slight degree. Although this idea is worthy of being borne in mind, Dr. Hall lays more stress upon it than it is perhaps entitled to.

The examination of the blood, or hæmatology, has also been made a guide in investigating the character of existing morbid actions; and it has led to most interesting results. Investigations of the quality of the blood have, indeed, led to a refutation of the idea once prevalent, that inflammation is necessarily present in every case of fever. In the healthy condition, the blood contains in every thousand parts, three of fibrin. In the experiments of MM. Andral and Gavarret it was found, that this constituent is increased by inflammation; and that its augmentation bears a ratio to the degree of existing inflammatory action. Thus, in mild cases it was found to rise to five parts, in severe ones to ten and a half, and in the medium to seven parts in the thousand. This being proved by repeated experiments, it was applied to determine the truth of the proposition, that in all cases ot fever, inflammation of some organ exists, But in continued fever it was found, that there was either no difference, or a diminution, of the fibrinous element. When, however, inflammatory action occurred in the course of fever, an immediate augmentation of fibrin was the consequence.

The increase of the proportion of the fibrinous element to that of the red globules is appreciated by the appearance of the blood, which is covered by the buffy coat. Although, however, the fibrinous element is increased by inflammation, the same circumstance may occur in other conditions of the system. It is noticed, indeed, in two opposite states—in high inflammation, and in chlorosis—a disease, which is benefited by the administration of large amounts of iron.

[The Professor here exhibited a small quantity of blood drawn from the arm of a chlorotic female; which presented a slight evidence of the buffy coat: he also pointed out the fact, that the watery parts of the blood, in such cases—as exhibited by the specimen—predominate. The coagulum was proportionally small and loosely cohering, instead of being firm and cupped, as in inflammation.]

Sometimes, valuable indications may be derived from examination of the secretions. A good exemplification of this is exhibited in granular disease of the kidney, as pointed out about seventeen years ago by Dr. Bright. The urine of a healthy person contains no evidences of albumen, but in this affection a very considerable quantity of the principle is at times secreted by the kidney. Albumen may be detected in urine by the addition of nitric acid which coagulates it. This appearance may, however, be deceptive, inasmuch as the acid may have decomposed certain salts of the urine, and the deposit may be mistaken for albumen. To prevent mistakes, heat should be applied to another portion of the same u ine; and if it produces coagulation, the inference is, that the albuminous principle is present, and the albuminuria leads to the suspicion that the case is one of granular disease of the kidney. As in other cases, however, this symptom must be conjoined with others before the existence of the affection can be positively proved.

The professor, after making these remarks, exhibited the effects of the above mentioned reagents on urine from a patient labouring under Bright's disease; and then presented before the class several interesting pathological specimens—the nature of which he demonstrated. Among these was one exhibiting the granular disease of the kidney just alluded to. The patient, a man aged 32, had passed considerable quantities of albuminous urine, and was affected with general dropsy, the fluid of which had distended the cellular tissue almost tô bursting,—the tissue of the scrotum and penis having contained about half a gallon of fluid. No other organ presented any morbid appearance except the kidney, which was slightly granulated, the tubular portion being healthy, and only the cortical involved.

The second specimen, from a man 27 years of age, exhibited the appearances presented by what is, in common parlance, termed "whisky liver"—the yellow or biliary element being hypertrophied, so that the appearance of the organ contrasted greatly with that presented by the liver of the other individual, in which there was congestion of the venous system. There was also very great increase of the natural adipose matter. This augmentation of the adipose matter of the liver is stated by Louis to be present in a third part of the livers of patients dying from pulmonary disease. The whisky liver, as the name imports, is found in those who drink largely of spiritous liquors. How the liver should have its nutrition modified in such cases is readily comprehensible. The alcohol enters the portal vessels which rise from the stomach, and is conveyed in a state of concentration to the liver, where, by exciting continually, it gives rise to nutritive aberration. This fatty condition of the organ may be detected by the greasy streak it gives to the scalpel when passed into its substance. [The appearance presented by the knife was shown to the class.]

The last specimen exhibited was one of a lung filled with tubercles, having a small cavity at the summit. The left lung was nearly in its healthy state. The Professor, by percussing the two lungs, demonstrated audibly to the class the difference between the clear sound rendered by the healthy lung, and the dull sound of solidification. The patient's death was caused immediately, by an attack of pleuro-pneumonia—and the lecturer remarked, that his attention at one of his visits was drawn to the presence of pneumonia in the case by the pungent heat of the surface, which an excellent observer—Dr. Stokes—considers as the most invariable and conclusive diagnostic. On percussion, the extensive consolidation which proved to be partly caused by tuberculosis and partly by pneumonia—was perceptible.

This specimen was from the same individual as the whisky liver.

The lecturer concluded by stating, that on future occasions he would endeavour so to select his cases at the Hospital, as to be elucidative of the principles taught by him during the week at the Jefferson Medical College.

